# Facile continuous process for gas phase halogen exchange over supported alkyl phosphonium salts

**DOI:** 10.1039/c7ra12579h

**Published:** 2018-01-12

**Authors:** Priti Sharma, Yoel Sasson

**Affiliations:** Casali Center of Applied Chemistry, Institute of Chemistry, The Hebrew University of Jerusalem Jerusalem 91904 Israel priti.sharma@mail.huji.ac.il

## Abstract

Chloride–bromide halogen exchange was realized when a mixture of an alkyl chloride and an alkyl bromide were reacted over a supported molten alkyl phosphonium catalyst. Conversion was found to be near equilibrium in a tubular flow reactor at 150 °C and 1500 GHSV. The catalyst was prepared by impregnation of alumina or silica support and found to be highly stable for relatively long periods of time. A pathway for the catalytic cycle is proposed.

## Abstract 2

Chloride–bromide halogen exchange was realized when a mixture of an alkyl chloride and an alkyl bromide were reacted over a supported molten alkyl phosphonium catalyst.

## Graphical abstract

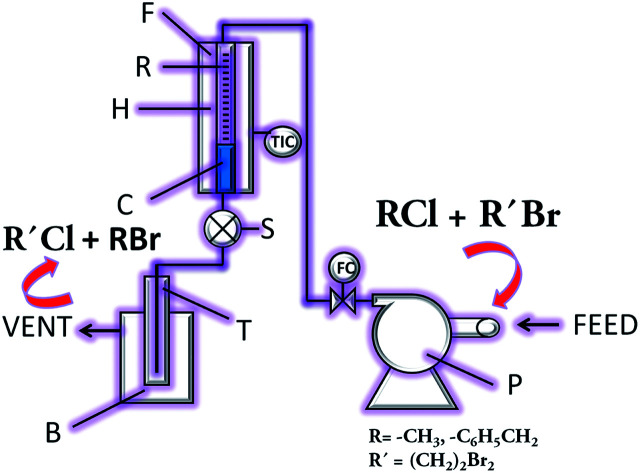

## Introduction

The halogen exchange of alkyl and aryl halides is a fundamental reaction in pharmaceutical and agrochemical industries.^1–4^ The alkyl and aryl halide functional groups can readily be used in a wide range of cross-coupling reactions,^5^ organometallic reagents,^6^ physical and biological properties modification of aromatic rings^7,8^ and generation of free-radical intermediates precursor.^1,9^ Gas phase halogen exchange is mainly used for preparing alkyl bromides or iodides, which are more reactive and can be readily employed in numerous transformations.^9–12^ For halogen exchange reaction, Finkelstein reactions^13^ have been reported specifically along with alkyl lithium,^14^ Grignard reagents,^15^ and copper-catalyzed alkylation for the conversion of alkyl chlorides to the corresponding bromides or iodides.^16–18^ However a large excess of the metal halide makes the Finkelstein reaction procedure uneconomical at industrial level.^19,20^ Few groups reported the use of ionic liquids for the halogen exchange reactions.^21–23^ Others exhibited phase transfer catalysts (PTC) to accelerate halogen exchange between metal salts and alkyl halides in organic phase. Nonetheless, these reactions still suffer from the disadvantage of reversibility, with the demand of large excess donor halide salt for obtaining significant yields of the alkyl halide product.^24–27^

In the same context; halogen exchange reactions between alkyl bromides and alkyl chlorides were homogeneously catalyzed by tertiary amines at 110 °C.^28,29^ Yonovich and Sasson studied bromide–chloride exchange between alkyl halide and metal halide salts under phase transfer conditions.^30^ These authors have observed that calcium bromide can be effectively used as brominating agent for primary alkyl halides in the presence of lipophilic quaternary ammonium salts as phase transfer catalysts. Sasson *et al.* stated that role of metal cation in solid liquid phase transfer catalysis conditions was much more critical than that of the anions which were generally considered the main effect in phase transfer reactions. Further in an economic approach Sasson and his coworkers effectively demonstrated the use of phase transfer catalyst in halogen exchange reaction at industrial level and laboratory-scale to convert inorganic bromide salts from the respective chloride salts by reaction with 1,2 dibromoethane.^31^ The described simple technique avoids the corrosive and exothermic conditions of the conventional processes.^32^

In continuations of the same study in the present work; we tried to establish a protocol for halogen exchange reaction as a convenient way to prepare a desired alkyl halide from corresponding alkyl halides using readily available and cheap alkyl phosphonium salts on supported solid phase (alumina or silica) for continuous gas phase process.

In the present study halogen exchange reaction between alkyl chloride and alkyl bromide substrates in the gas phase is reported by using supported molten quaternary phosphonium salts as catalysts at temperature between 140–160 °C and at atmospheric pressure. Quaternary phosphonium salts are known to be more resilient to high temperature in comparison with the corresponding ammonium salts. 1,2-Dibromo (EDB) was used as the brominating agent, since it is an available and cheap source of bromine.

## Experimental

Chemicals used in the experiments: alkyl phosphonium bromide, dichloromethane (Merck), alumina (Strem chemicals), silica (Alfa products), tetrabutyl phosphonium bromide (Aldrich), hexadecyl tributyl phosphonium bromide (Fluka). Ethylene dibromide and alkyl chlorides (Aldrich) were applied as received without further purifications.

### Catalyst preparation

The catalyst was prepared by impregnation method. A solution containing the appropriate amount of the alkyl phosphonium bromide in a suitable amount of dichloromethane (Merk) was prepared. The alkyl phosphonium solution was added to the alumina (Strem chemicals) or silica (Alfa products) used as the supports. The mixture was evaporated to dryness at 50 °C in a flash evaporator and dried in air at 120 °C. Catalysts containing between 2% and 20% by weight of phosphonium salt were prepared by this method. The tetrabutyl phosphonium bromide (Aldrich) and the hexadecyl tributyl phosphonium bromide (Fluka) were used as obtained from suppliers.

### Procedure

**Fig. 1:**
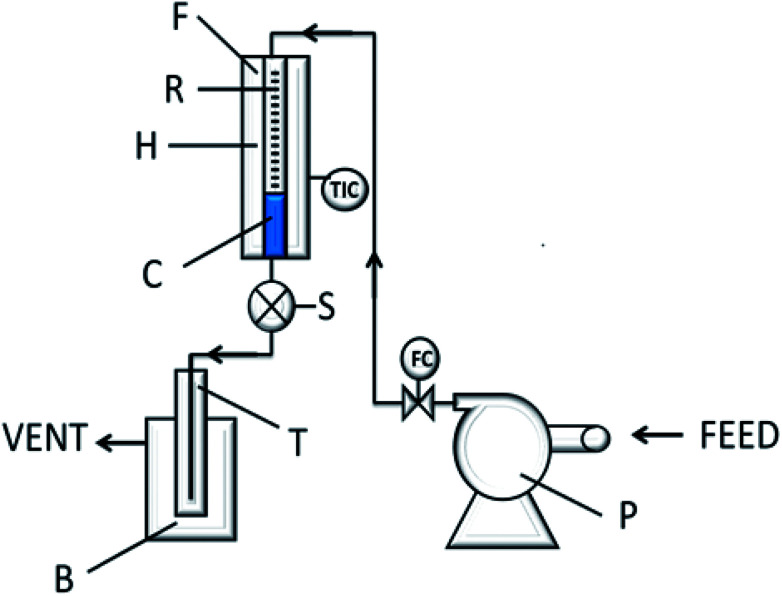
Flow diagram of the reaction system. F = furnace, R = pyrex reactor, H = preheater, C = catalyst bead, S = sampling device, T = trap, B = cold batch, P = peristaltic pump.

The experimental set up is described in Fig. 1. In a typical run a mixture of an alkyl chloride and 1,2 dibromoethane (EDB) were introduced into the reactor by a peristaltic pump. The alkyl halides were gasified over glass beads before reaching the catalyst. The resulting mixture was collected in a cold trap at the outlet of the reactor and analyzed by GLC. Typical experiments were carried out at temperature between 140–160 °C, atmospheric pressure and GHSV between 1500–2000. The EDB (Riedel) used as the brominating agent and the alkyl chlorides (Aldrich or Fluka) were used as received.

## Results and discussion

The halogen exchange reaction between alkyl halides is an equilibrium reaction. In the present study, when a mixture of alkyl chloride and EDB were reacted in the gas phase in the presence of an alkyl phosphonium salt catalyst supported on alumina or silica, a mixture of alkyl halides was obtained. Molar conversions obtained per path were near to equilibrium conversions and were typically between 50–80%, depending on the nature of the alkyl halides.

**Fig. 2:**
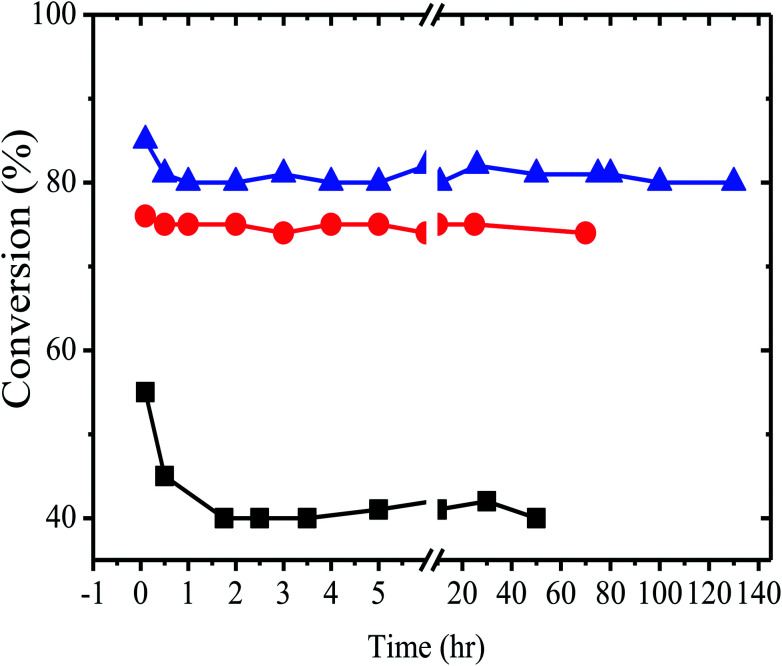
Activity of the alkyl phosphonium catalysts as a function of time at 150 °C, (a) (▲) = 10% tetrabutyl phosphonium/Al_2_O_3_, (b) (●) = 10% hexadecyl tributyl phosphonium/Al_2_O_3_, (c) (■) = 10% tetrabutyl phosphonium/SiO_2_. At 150 °C, 1 atm and 1500 GHSV. 1 : 1 molar mixture of reactants (EDB + EDC).

Fig. 2 shows the behavior of the reaction of ethylene dichloride with ethylene dibromide at 150° over three different catalysts. 10% w/w tetrabutylphosphonium bromide on alumina, 10% w/w hexadecyltributylphosphonium bromide on alumina and 10% w/w of tetrabutylphosphonium bromide on silica. Al_2_O_3_ supported catalysts (∼85%) shows good conversion rather than the SiO_2_ (∼55%) supported catalysts shown in Fig. 2. However Al_2_O_3_ or SiO_2_ heterogeneous support alone do not show any conversion but with combination of tetrabutyl phosphonium or hexadecyl tributyl phosphonium with Al_2_O_3_ enhanced halogen exchange reaction multiple folds (upto 80% product conversion). Probably due to Al_2_O_3_ acidic group interaction with phosphonium bromide catalyst.^33,34^ We may conclude that the equilibrium conversion under these conditions is above 80%.

Typical results obtained over 10% (weight) tetrabutyl phosphonium bromide supported on the alumina catalyst are presented in Table 1, when molar mixture of 1 : 1 of the reactants were used. The conversion is easily increased by using an excess of EDB.

**Table 1:** Bromide chloride exchange in the gas phase over 10% (C_4_H_9_)_4_PBr/Al_2_O_3_ catalyst at 150 °C, 1 atm and 1500 GHSV. 1 : 1 molar mixture of reactants

RCl + R′Br → R′Cl + RBr		
Entry	Reaction	Conversion per path (% mol)
1.	C_6_H_5_CH_2_Cl + (CH_2_)_2_Br_2_ → C_6_H_5_CH_2_Br + Br(CH_2_)_2_Cl + (CH_2_)_2_Cl_2_	70
2.	CH_3_(CH_2_)_5_Cl + (CH_2_)_2_Br_2_ → CH_3_(CH_2_)_5_Br + Br(CH_2_)_2_Cl + (CH_2_)_2_Cl_2_	67
3.	CH_3_(CH_2_)_4_Cl + (CH_2_)_2_Br_2_ → CH_3_(CH_2_)_4_Br + Br(CH_2_)_2_Cl + (CH_2_)_2_Cl_2_	62
4.	CH_3_(CH_2_)_3_Cl + (CH_2_)_2_Br_2_ → CH_3_(CH_2_)_3_Br + Br(CH_2_)_2_Cl + (CH_2_)_2_Cl_2_	51
5.	CH_2_Cl_2_ + (CH_2_)_2_Br_2_ → CH_2_CIBr + Br(CH_2_)_2_Cl + CH_2_Br_2_ + (CH_2_)_2_Cl_2_	∼5
6.	BrCH_2_CH_2_Br + Cl(CH_2_)_2_Cl → 2BrCH_2_CH_2_Cl	80

**Fig. 3:**
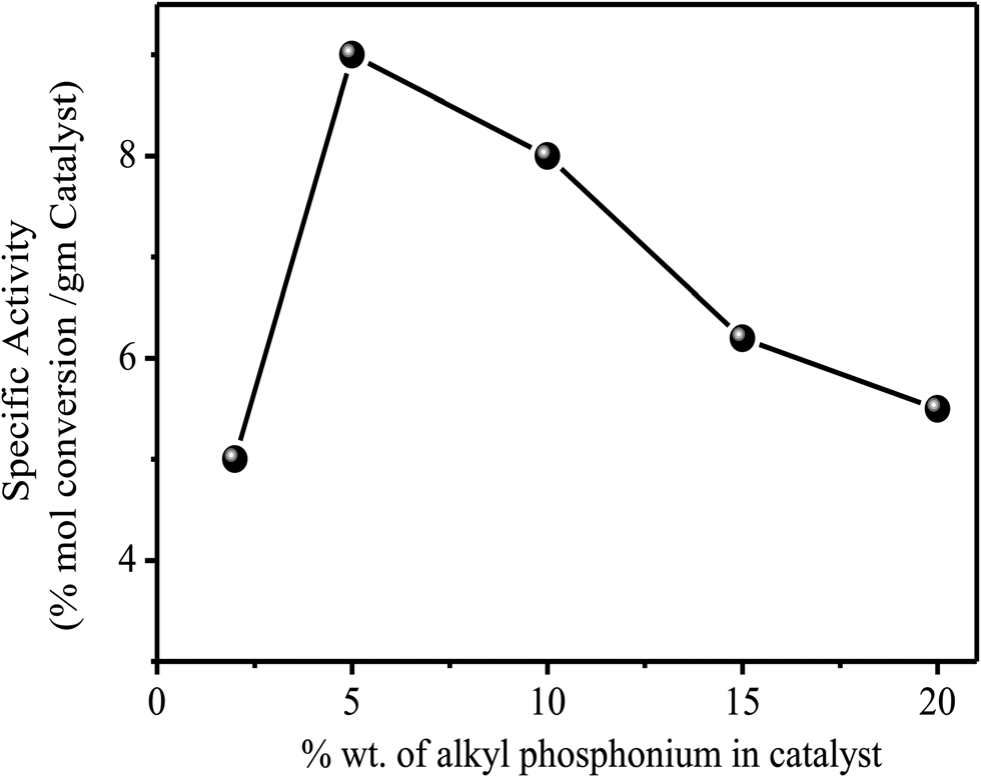
Influence of tetrabutyl phosphonium load on the activity of the catalyst in the reaction of EDB + EDC at 150 °C. At 150 °C, 1 atm and 1500 GHSV. 1 : 1 molar mixture of reactants.

The catalysts were found to be highly stable, giving almost the same rate of reaction over relatively long periods of time. These results are clearly illustrated in Fig. 2, which describes the activity of the catalysts over a period of more than 100 h. As expected from the theory of SLPC^29^ there is an optimal value of the liquid loading on the catalyst. Table 2 and Fig. 3 presents the summarized results of a series of experiments in which the liquid loading was varied. From these results the effect of the liquid load is clearly observed. For example, it can be realized that for the TBP/Al_2_O_3_ catalyst the optimal liquid load is in between 5–10% (weight).

**Table 2:** Influence of the liquid loading on the activity of catalyst (load given in % weight of catalyst)

Entry	Catalyst	Specific activity (% mol conversion/gm catalyst)
**C**_**16**_ **H**_**33**_ **[CH**_**3**_ **(CH**_**2**_ **)**_**3**_ **]**_**3**_ **PBr**		
1.	10%/Al_2_O_3_	7.8
2.	15%/Al_2_O_3_	6.7
3.	20%/Al_2_O_3_	5.1

**[CH**_**3**_ **(CH**_**2**_ **)**_**3**_ **]**_**4**_ **PBr**		
1.	2.5%/Al_2_O_3_	4.8
2.	5%/Al_2_O_3_	8.8
3.	10%/Al_2_O_3_	8.4
4.	15%/Al_2_O_3_	6.3
5.	20%/Al_2_O_3_	5.4
6.	5%/SiO_2_	5.2
7.	10%/SiO_2_	4.3

Exchange reaction is(CH_2_)_2_Cl_2_ + (CH_2_)_2_Br_2_ → 2(CH_2_)_2_BrCl1 : 1 molar mixture at 150 °C, 1 atm and 1500 GHSV.

Additional experiments are necessary to obtain accurate values of the liquid loading optimum. It is interesting to point out that neither the SiO_2_ nor the Al_2_O_3_ used as the supports were active in this reaction, when used unloaded. The alumina gives very small conversions for the exchange reaction (<5%) and mainly products of elimination reactions were detected. Therefore from Table 2 it is very clear that support (SiO_2_ or Al_2_O_3_) is not playing active role in halogen exchange reaction. A possible pathway for the exchange reaction over the alkyl phosphonium catalyst is summarized in the following scheme.

1 RCl + QBr → RBr + QCl

2 R′Br + QCl → R′Cl + QBr

The overall catalytic cycle is summarized by the reaction;

3 RCl + R′Br → RBr + R′Cl

**Fig. 4:**
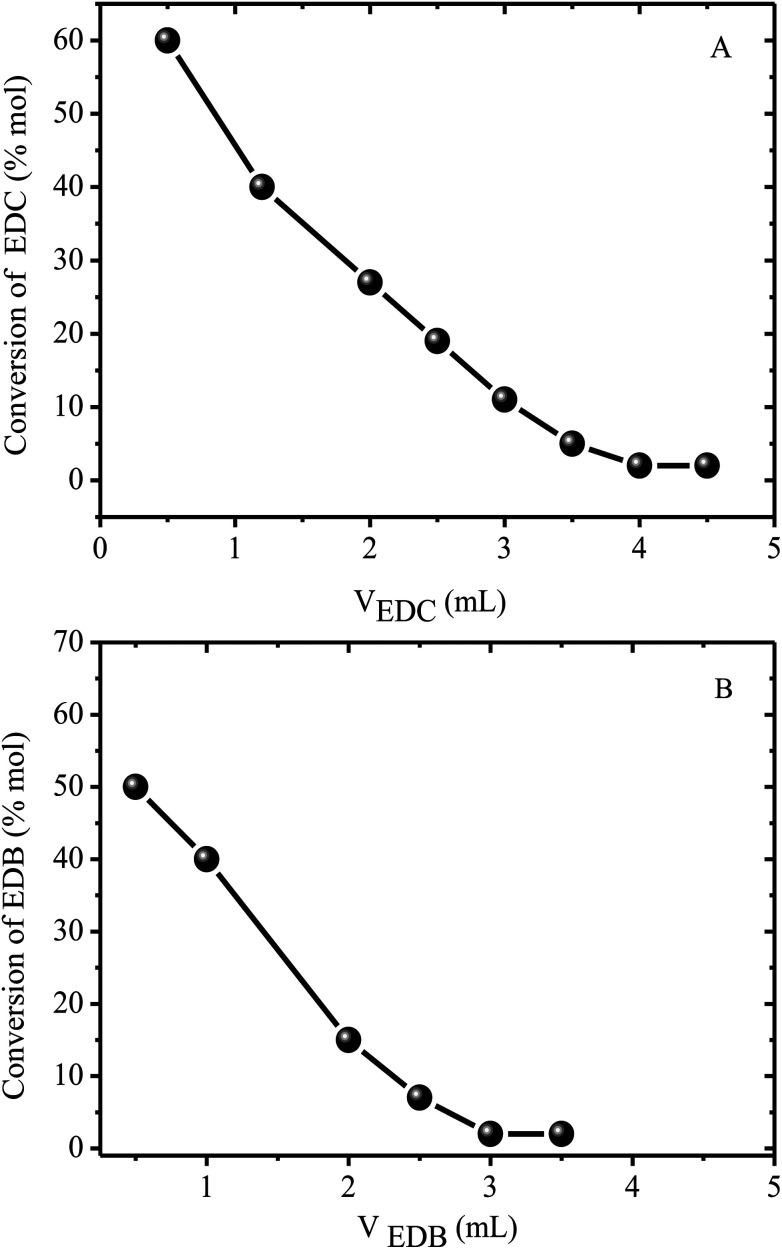
Reaction of the alkyl halides with the alkyl phosphonium catalyst at 150 °C. (A) = reaction of 1,2 dichloro ethane (EDC) with tetrabutyl phosphonium bromide (reaction (1)). (B) = reaction of 1,2 dibromoethane (EDB) with tetrabutyl phosphonium chloride (reaction (2)).

The proposed catalytic sequence is supported by the results obtained in a series of experiments (Fig. 4) in which the alkyl phosphonium bromide catalyst was reacted first with the alkyl chloride converting the alkyl phosphonium bromide into the chloride and producing the respective alkyl bromide (reaction (1)). The alkyl phosphonium chloride was then reacted with the alkyl bromide according to reaction (2).

The reaction between 1,2 dichloro ethane (EDC) and 1,2 dibromoethane (EDB) was used to illustrate the proposed mechanism because of its relative simplicity giving only one possible reaction product according to the equation;

4 BrC_2_H_4_Br + ClC_2_H_4_Cl → 2BrC_2_H_4_Cl

**Fig. 5:**
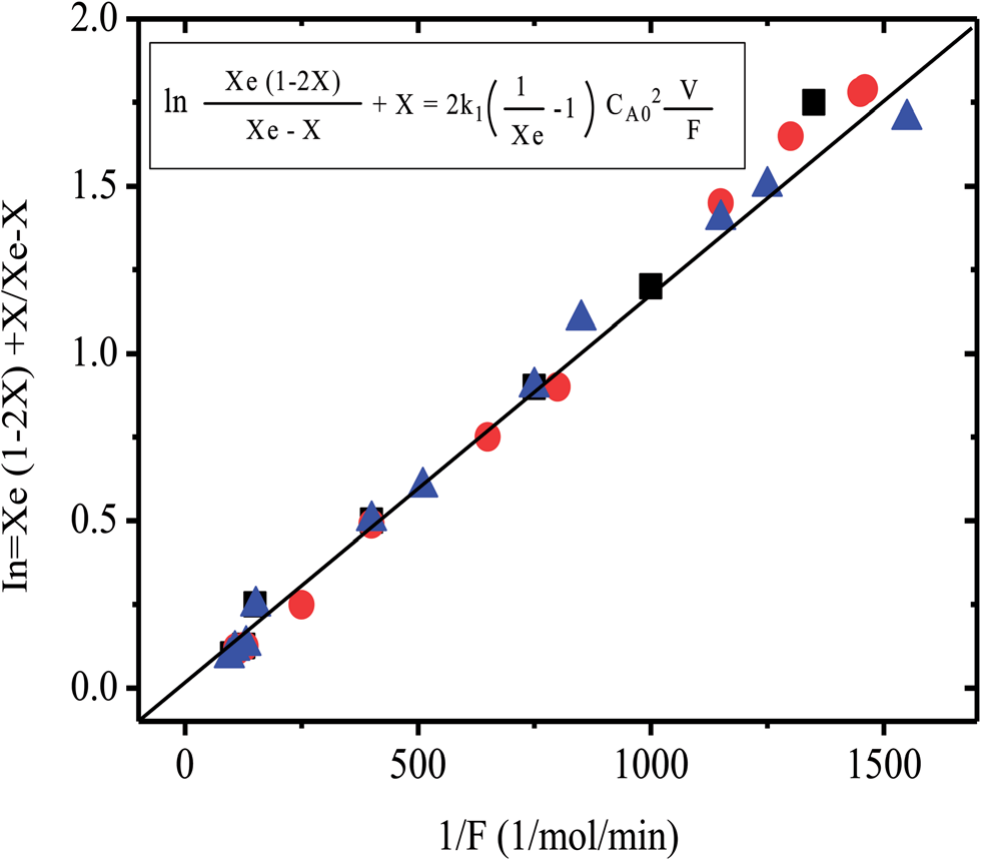
Plot of variables for the second order reversible kinetics for the halide exchange reaction (4), over 10% tetrabutyl phosphonium/Al_2_O_3_ at 148 °C. Symbols are for different experiments.

Additionally evidence supporting the proposed catalytic cycle could be obtained from preliminary kinetic investigation carried out in this laboratory. From the results obtained, the reaction seems to follow a second order reversible kinetics, according to the equation (Fig. 5);
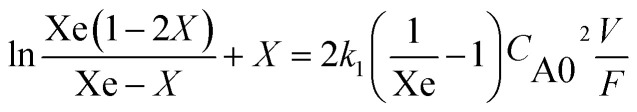
Xe = equilibrium conversion, *k*_1_ = second order rate reaction, *V* = reactor volume, *F* = molar flow rate, *C*_A0_ = concentration of A at the entrance of reactor, *X* = conversion.

A plot of this kinetic equation using the experimental results is presented in Fig. 5 (second order reversible kinetics). The equilibrium conversion required was estimated by method described elsewhere.^35^ However a more detailed kinetic investigation should be made before conclusive evidence could be obtained. Such a kinetic investigation is under study in this laboratory in order to determine the accurate rate equation of this reaction.

## Conclusions

Herein we demonstrated an efficient equilibrium conversion method for halogen exchange (chloride to bromide) using molten alkyl phosphonium catalyst without any additional material usage in multiple cycles in a long continuous flow. Alkyl phosphonium catalyst reactivity screened under various optimization parameters *viz*; loading (2–20%), supported over (silica or alumina) for halogen exchange reaction. The silica and alumina based phase-transfer catalyst easily regenerated and reused several times without loss of their activity. The products of the exchange reactions could be simply separated by distillation under normal pressure.

## Conflicts of interest

There are no conflicts of interest in the present research work.

## Supplementary Material
